# Revised algorithm for heparin anticoagulation during continuous renal replacement therapy

**DOI:** 10.1186/s13054-015-1099-y

**Published:** 2015-10-27

**Authors:** Helen Dickie, Linda Tovey, William Berry, Marlies Ostermann

**Affiliations:** Department of Critical Care Medicine, King’s College London, Guy’s & St Thomas’ NHS Foundation Hospital, London, SE1 7EH UK

Premature clotting of the circuit is the most common reason for unplanned interruptions in renal replacement therapy (RRT) and discrepancies between the prescribed and delivered dose of RRT [1]. The recent guideline from the Kidney Disease Improving Global Outcomes (KDIGO) working group recommended citrate as the first-line anticoagulant for continuous renal replacement therapy (CRRT) [2]. For patients with contraindications to citrate, either unfractionated or low molecular weight heparin was recommended.

Worldwide, unfractionated heparin is the most commonly used anticoagulant to maintain circuit patency [1]. The potential risks including bleeding and heparin-induced thrombocytopenia are well known. In 2010, we designed an algorithm to enable the nursing staff to manage heparin during CRRT effectively and safely [3]. In response to new knowledge and changes in clinical practice, we have revised the algorithm (Fig. 1).

**Fig. 1:**
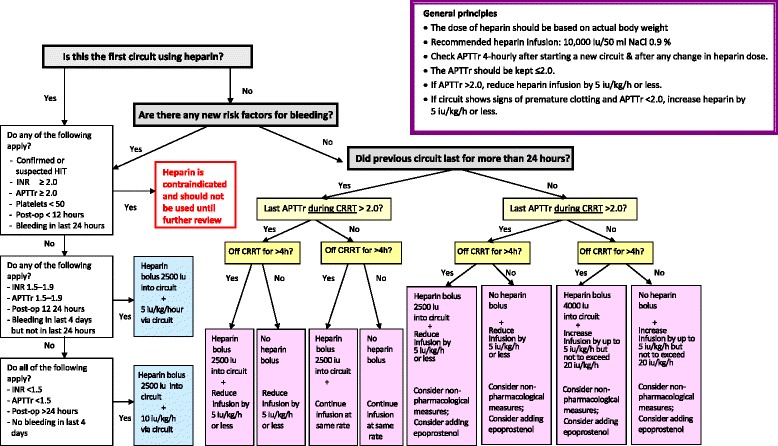
Algorithm for heparin anticoagulation during CRRT. *APTTr* activated partial thromboplastin time ratio, *CRRT* continuous renal replacement therapy, *HIT* heparin-induced thrombocytopenia, *INR* international normalised ratio

The main principles of the algorithm are maintained as follows:

1. The indication for heparin should be reviewed daily. Heparin should be avoided in patients with an increased bleeding risk.
2. Unfractionated heparin is administered via the circuit unless the patient needs systemic anticoagulation for other reasons.
3. The dose of heparin is based on actual body weight.
4. Dosing of heparin is “individualised” depending on the patient’s risk of bleeding and previous circuit life.
5. There is no target activated partial thromboplastin time ratio (APTTr) but APTTr ≤2 is maintained to prevent overanticoagulation.
6. Nonpharmacological methods should be considered regularly to maintain circuit patency.

The main changes to the algorithm are the following:

1. We previously suggested adding heparin to the circuit priming solution before the blood is in contact with plastic surfaces to coat the surfaces of the filter membrane and circuit tubing. It has since been brought to our attention that a randomised controlled cross-over study in 11 patients on CRRT showed no beneficial effect of heparin rinse on the thrombogenicity of the circuit, complement activation or blood leukocyte counts [4]. Therefore, instead of using 10,000 IU heparin in 1 l of 0.9 % saline to prime a circuit with total volume ~270 ml, we suggest administering a bolus of 2500 IU heparin on connection.
2. Following the withdrawal of activated protein C [5], a reference to this drug has been removed from the algorithm.

The systemic effects of heparin are a potential drawback of CRRT. We hope that our proposed algorithm reduces this risk and allows effective and safe anticoagulation.

## Abbreviations

APTTr: Activated partial thromboplastin time ratio
CRRT: Continuous renal replacement therapy
KDIGO: Kidney Disease Improving Global Outcomes
RRT: Renal replacement therapy
